# Migration of Health Workers and Its Impacts on the Nigerian Health Care Sector: Protocol for a Scoping Review

**DOI:** 10.2196/62726

**Published:** 2025-01-30

**Authors:** David Omiyi, Ebenezer Arubuola, Marcus Chilaka, Md Shafiqur Rahman Jabin

**Affiliations:** 1 Faculty of Health Studies School of Allied Health Professions and Midwifery University of Bradford Bradford United Kingdom; 2 Department of Medicine and Optometry eHealth Institue Linnaeus University Kalmar Sweden; 3 Faculty of Health Studies School of Nursing and Healthcare Leadership University of Bradford Bradford United Kingdom

**Keywords:** training and education, health policy, healthcare workforce, policy interventions, socio-political factors, political instability, workforce capacity

## Abstract

**Background:**

Health worker migration from Nigeria poses significant challenges to the Nigerian health care sector and has far-reaching implications for health care systems globally. Understanding the factors driving migration, its effects on health care delivery, and potential policy interventions is critical for addressing this complex issue.

**Objective:**

This study aims to comprehensively examine the factors encouraging the emigration of Nigerian health workers, map out the effects of health worker migration on the Nigerian health system, document the loss of investment in health training and education resulting from migration, identify relevant policy initiatives addressing migration, determine the effects of Nigerian health worker migration on destination countries, and identify the benefits and demerits to Nigeria of health worker migration.

**Methods:**

This study will follow the Joanna Briggs Institute methodology. A search strategy will retrieve published studies from MEDLINE, CINAHL, Embase, Global Health, Academic Search Premiere, and Web of Science. Unpublished studies will be sourced from dissertations and theses. A comprehensive search will involve keyword scans and citation searches. Exclusion criteria will filter out irrelevant studies, such as studies unrelated to the international migration of health workers and non-English language studies. A total of 2 independent reviewers will screen the titles and abstracts and then review the full text. Data will be extracted from the included studies using a data extraction tool developed for this study. The study selection process will be shown using a PRISMA-ScR (Preferred Reporting Items for Systematic Reviews and Meta-Analyses extension for Scoping Reviews) flowchart. While the traditional risk of bias assessments is not applied to scoping reviews, the quality of included studies will be evaluated based on methodological transparency.

**Results:**

The process of selecting studies will be shown using a PRISMA ScR flowchart, and information gathering will be done through a charting table that has been prepared in advance. We plan to collect data from January 2025 to March 2025 and present the results to examine publication patterns and study details. The final summary is expected to be released by the summer of 2025. It will provide an in-depth look at how health worker migration impacts the health care sector in Nigeria.

**Conclusions:**

This study holds immense potential to contribute to understanding health worker migration from Nigeria and inform policy and practice interventions to address its challenges. By synthesizing existing evidence, the scoping review will guide future research and policy efforts to mitigate the adverse effects of migration on health care systems and workforce sustainability. Furthermore, the results will aid in recognizing deficiencies in the existing literature; this will offer a defined path for specific policy measures and methods to retain health care workers effectively and thus support the sustainability of health care systems.

**International Registered Report Identifier (IRRID):**

PRR1-10.2196/62726

## Introduction

### Overview

Nigeria is the most populous country in Africa and has one of the largest supplies of health workers on the continent; however, the country has been particularly affected by the migration issue [1]. It is estimated that 20,000 of the 72,000 Nigerian physicians educated in Nigeria were practicing abroad in 2008 [2]. This number represents 28% of the physician workforce trained in Nigeria and is significantly higher than the proportion for any other African country [3]. As a result, Nigeria has a fragile health system that is unable to effectively deliver quality health services to its populace. This includes increased workload for the remaining health workers, decreased access to health care services, and compromised quality of care [4-6]. Furthermore, Nigeria invests substantial resources in training its health workforce, yet many of these trained professionals ultimately migrate to seek better opportunities abroad. Documenting the loss of investment in health training and education due to health worker migration is essential for assessing the economic and human resource implications for Nigeria's health care system [7].

The global crisis in human resources for health has been described as one of the most pressing issues facing the health sector and is now widely acknowledged as a global priority [8]. Addressing the shortage and maldistribution of health workers, including policy dialogue and coordinated action in the area of international migration of health workers, is deemed necessary to avert a long-term global health workforce crisis, which would be detrimental to the attainment of the health-related Sustainable Development Goals and Universal Health Coverage, and ultimately to global health [9]. Policy initiatives have been proposed to address health worker migration in Nigeria, including retention schemes such as offering competitive salaries and benefits, incentive programs like providing opportunities for professional development, and bilateral agreements to regulate the recruitment of Nigerian health workers by foreign countries. Evaluating the effectiveness and impact of these policies is critical for policy makers and stakeholders to develop evidence-based strategies to mitigate the adverse effects of health worker migration [10]. In addition, understanding the effects of Nigerian health worker migration on destination countries is vital for promoting ethical recruitment practices and fostering global health workforce planning. While migration poses challenges for the Nigerian health care system, it also generates remittances, fosters knowledge exchange, and promotes international engagement in health care development initiatives [11].

The migration of highly skilled health workers from developing to developed countries has been cited as contributing to shortages of qualified health workers in many African and Southeast Asian countries. In relation to the Nigerian context, there is evidence that Nigeria has experienced high levels of emigration of health professionals, where the United Kingdom, United States, and Canada are among the leading destination countries [12]. It is important to understand the journeys, distribution, and characteristics of migrant health workers, with the aim of identifying specific policy measures to enhance positive impacts and mitigate negative impacts of migration on health systems in source and destination countries [9,13,14]. In summary, this scoping review protocol seeks to provide a comprehensive overview of health worker migration and its impacts on the Nigerian health care sector. By addressing these issues, the review will inform evidence-based policies and interventions to optimize the management of health worker migration and strengthen the resilience of Nigeria’s health care system.

A preliminary search of Campbell’s systematic reviews, the Cochrane Database of Systematic Reviews, PROSPERO (International Prospective Register of Systematic Reviews), and Joanna Briggs Institute (JBI) evidence synthesis was conducted. Interestingly, no current or underway systematic reviews or scoping reviews on the topic were identified, highlighting the unique contribution of this study.

### Aim and Review Questions

The primary purpose of this study is to establish a better understanding of how migration affects individual developing nations. This understanding will pave the way for new policies that can better manage the recruitment and retention of health professionals, thereby minimizing any negative effects caused by migration. Importantly, this study also holds the potential for positive outcomes, such as increased skill and knowledge within the sector, which can significantly benefit the health care system.

This review will seek to identify the breadth and depth of the available literature, including studies on the causes and consequences of health worker migration, the effectiveness of policy interventions, and the experiences of health workers and their families. This will provide an opportunity to identify research gaps and inform future research and policy decisions. At this preliminary stage in conducting the scoping review, the authors have also found that this type of review is best suited to the resources and time available. Specifically, the review questions are as follows: (1) What factors contribute to the emigration of Nigerian health workers? (2) How does health worker migration impact the Nigerian health system, including its strengths and weaknesses? (3) What effective strategies should be used to promote the retention of skilled health workers within the Nigerian health care system?

## Methods

### Overview

Following the JBI methodology [15], the study will conduct a comprehensive search across multiple databases to find relevant studies. Data from these studies will be systematically extracted, organized, and analyzed, ensuring a reliable and comprehensive review, with findings presented in a clear and descriptive format.

### Search Strategy

The scoping protocol, a detailed plan outlining the methods and search strategy for the scoping review, will initiate with an initial limited search of databases, focusing on analyzing text words in titles, abstracts, and index terms. This will be followed by a comprehensive 2-way search strategy across multiple databases, including MEDLINE, CINAHL (EBSCO), Embase, Global Health, Academic Search Premiere, and Web of Science (Table 1). Unpublished studies will be sourced from dissertations and theses. The search will involve specified keywords (eg, emigration, immigration, and health workers) and free keywords (eg, brain drain and migration). Additional strategies, such as citation and chain searches, will be used to enhance search completeness. Exclusion criteria will filter out studies unrelated to the international migration of health workers or not centered on the Nigerian health care sector. Only English-language studies from an appropriate date range will be considered, aligning with the review’s scope and context. Finally, hand-searching of reference lists from relevant primary studies or review articles will be conducted. To address potential publication bias, the review will include measures such as searching for gray literature, unpublished studies, and reports that may contain negative or nonsignificant findings. This approach will help ensure a more comprehensive and balanced understanding of the topic. The scoping protocol plays a crucial role in ensuring the systematic and rigorous conduct of the scoping review, and its adherence will be reported in the final scoping review.

**Table 1:** Search strategy on databases.

Participant, context, and concept scheme	Number	Search string	MEDLINE (March 28)	CINAHL	Web of Science	Embase	Global Health	Academic Search Premier
International migration AND its impacts on the Nigerian healthcare sector	1	emigration OR immigration OR health workers OR skilled healthcare workers OR health professionals OR healthcare professionals OR healthcare personnel	339,025	119,968	349,710	22,039	12,876	3789
International migration AND its impacts on the Nigerian healthcare sector	2	brain drain OR migration OR healthcare workers	387,827	43,054	851,446	9098	456	1789
	3	#1 AND #2	19,789	11,006		2004	55	2908
Migration AND its impacts on the Nigerian healthcare sector	4	Nigeria OR Nigerian	41,946	7366	4540	455	34	45
Combined	5	#3 AND #4	292	113		23	12	9
Filters	6	English, from 2001 to date (excluding Magazine and book chapters)	33	112		34	33	54

### Eligibility Criteria

This scoping review will incorporate the PCC (Population, Concept, Context) framework as a guide, ensuring a comprehensive and inclusive approach. We will thoroughly describe the characteristics of participants, concepts, and contexts alongside our search strategies, data extraction methods, analysis techniques, and result presentation formats. The eligibility criteria, designed to be comprehensive and inclusive, are detailed in Textbox 1. In addition, this review will involve iterative consultations with experts and stakeholders to refine the research questions and inclusion criteria.

**Inclusion and exclusion criteria.**

**Inclusion criteria:**

- Health workers migration from Nigeria.
- English.
- Studies from 2001 to 2024.
- Conference papers.
- Quantitative and qualitative evidence.
- Gray literature.
- Studies identifying policy initiatives aimed at addressing health worker migration in Nigeria.
- Research mapping out the impacts of health worker migration on the Nigerian health system.

**Exclusion criteria:**

- Meeting abstracts.
- Editorial materials.
- Book chapters.
- All other languages.
- Studies from 2001 and earlier.
- Studies not related to health worker migration from Nigeria.
- Literature not focused on the effects of health worker migration on the Nigerian health system.
- Articles lacking information on factors encouraging health worker emigration from Nigeria.
- Publications not discussing policy initiatives addressing health worker migration in Nigeria.

### Participants

This scoping review will include studies involving health workers who have migrated from Nigeria to other countries. The focus will be on health care professionals, including but not limited to doctors, nurses, midwives, pharmacists, radiographers, and laboratory scientists. In addition, individuals receiving health care services in Nigeria impacted by the migration of health workers will also be considered participants in this review.

### Concept

The primary concept under investigation is the migration of health workers and its impacts on the Nigerian health care sector. This includes examining factors contributing to health worker migration, such as motivating factors, as well as the consequences of migration on health care delivery, workforce dynamics, health care access, quality of care, and health outcomes in Nigeria.

### Context

The review will consider studies conducted regarding the Nigerian health care sector, including public and private health care facilities, hospitals, clinics, primary health care centers, and community health centers. Equally important, studies focusing on the health care systems of destination countries where Nigerian health workers have migrated will also be included to provide a comprehensive understanding of the context.

### Types of Sources

This scoping review will consider a variety of sources, including empirical research studies, policy documents, reports, and gray literature. Gray literature, which refers to nontraditional sources of information that are not published in commercial publications, can include conference papers, theses, government documents, and unpublished research. Specifically, quantitative studies such as surveys, cohort studies, and quantitative analyses of secondary data will be included. Qualitative research using methods such as interviews, focus groups, and case studies will also be considered. In addition, policy documents, government reports, organizational reports, and academic publications addressing the migration of health workers and its impacts on the Nigerian health care sector will be included in the review. For instance, a government report on the brain drain of health workers from Nigeria would be a relevant source for this review.

### Study or Source of Evidence Selection

Following the search, all identified citations will be collated and uploaded into EndNote (version 20; Clarivate Analytics), and duplicates will be removed. Following a pilot test, titles, and abstracts will then be screened by 2 or more independent reviewers for assessment against the inclusion criteria for the review. Potentially relevant sources will be retrieved in full, and their citation details will be imported into the JBI System for the Unified Management, Assessment, and Review of Information [16]. Two or more independent reviewers will thoroughly assess the full text of selected citations against the inclusion criteria. Reasons for the exclusion of sources of evidence in full text that do not meet the inclusion criteria will be recorded and reported in the scoping review. Any disagreements that arise between the reviewers at each stage of the selection process will be resolved through discussion or with an additional reviewer or reviewers. The results of the search and the study inclusion process will be reported in full in the final scoping review and presented in a PRISMA-ScR (Preferred Reporting Items for Systematic Reviews and Meta-Analyses extension for Scoping Reviews) flow diagram [17]. This process ensures the transparency and rigor of the study selection process.

### Data Extraction

To ensure the reliability and consistency of the data, 2 independent reviewers will meticulously extract data from each included study using a standardized data extraction form. The data extracted will include specific details about the participants, concept, context, study methods, and key findings relevant to the review questions. This thorough process guarantees the accuracy of our findings. A draft charting table will be developed as a data extraction tool. This charting table will also serve as the basis for data synthesis, and it will be modified and revised as necessary during the process of extracting data from each included evidence source. The purpose of this table is to provide a structured format for recording the extracted data, ensuring consistency and facilitating the analysis process. The synthesis will follow a narrative, descriptive approach, with identified patterns, gaps, and emerging themes categorized for further analysis. Modifications to the charting table will be detailed in the scoping review. Any disagreements that arise between the reviewers will be resolved through discussion or with an additional reviewer or reviewers. If appropriate, authors of papers will be contacted to request missing or additional data, where required.

## Results

The findings of this scoping review will be shared once we finish extracting the data as planned. Our goal is to finalize the data mapping and synthesis by March 2025. The review results will be organized to showcase a thorough overview of the data based on the main themes recognized during the review process. The table for charting will contain information like the publication year and country of the study as well as its objectives and methodology, along with details on the population studied and sample size considered in the research analysis process, including outcomes assessed and intervention duration when relevant. The final summary of results, which will detail the impact of health worker migration on Nigeria’s health care system, is anticipated to be released by the summer of 2025. The analysis will be finalized and submitted for publication in the autumn of 2025.

## Discussion

### Principal Findings

This scoping study will uncover insights into how health workers from Nigeria migrate and how it affects the country’s health care system. The results should highlight patterns in what drives their migration, such as reasons and job prospects along with working conditions, while also pointing out the difficulties caused by this shortage of health care workers in delivering services and ensuring access to health care. Furthermore, the review seeks to pinpoint areas where existing research lacks information, which can serve as a basis for studies and policy making decisions to address the consequences of health worker migration.

Dealing with health worker migration challenges is crucial to improving health care delivery. Like countries facing an aging population, Nigeria is also tackling both an aging populace and a scarcity of health care workers, which is straining the health care system [10]. Projections suggest a severe shortage in the health care workforce by 2038, underscoring the urgent need to address health worker emigration as a top priority [13]. This urgency should resonate with policy makers, health care administrators, researchers, and professionals involved in health care workforce management and policy development in Nigeria.

Recent studies on health worker migration in Nigeria shed light on the factors contributing to the departure of professionals from the country. For instance, some studies pointed out that sociopolitical factors such as political instability and insecurity, as well as professional factors like low wages and poor working conditions, are driving health worker emigration [9,14]. The findings underscore the potential of focused interventions to address issues and motivate health workers to remain in Nigeria, instilling a sense of optimism for the future of Nigeria’s health care system.

The consequences of health worker migration go beyond workforce shortages; they also impact the quality and accessibility of health care services in Nigeria. For instance, some research studies emphasized how health care delivery is strained and how health inequalities worsen due to the departure of professionals [12,18]. This should underline the gravity of the situation for policy makers, health care administrators, researchers, and professionals involved in Nigeria’s health care workforce management and policy development. Moreover, the issue of health workers leaving the country poses challenges to the health care systems’ long-term stability and ability to withstand pressures, highlighting the need for measures to retain and develop capacity [7].

Efforts to address these obstacles and encourage health professionals to stay within Nigeria’s health care system have been gaining traction. Research conducted by Ossai et al [19] Falase et al [3] emphasized the significance of overcoming hurdles and offering incentives to retain health workers in the country. Furthermore, the World Health Organization’s Global Code of Practice on International Recruitment of Health Personnel serves as a guideline for collaboration in tackling health worker migration and bolstering health care systems [20].

In summary, this review aims to focus on the reasons and effects of health care worker migration from Nigeria and sheds light on the challenges faced by the workforce and health care disparities in the country. Drawing comparisons with research will help put the findings into perspective by highlighting trends and new insights discovered. The study’s strengths lie in its methodology and thorough analysis of data; however, it may have limitations due to gaps in less-known sources. Moving forward, efforts should be made towards creating targeted policy recommendations and implementing strategies to mitigate the impacts of migration on the health care system. An effective dissemination strategy guarantees that the research outcomes are communicated to policy makers and experts in the field so they can contribute to solutions.

### Strengths and Limitations of the Study

The scoping review is designed to identify the gaps in the literature and recommend strategies for further research in this area. The methods used will provide us with an idea of how much research already exists in this domain and examine how research has been conducted on this topic [21]. In addition, the research team, with its extensive experience in different types of systematic reviews, brings a wealth of knowledge and expertise in this specific field, making it well-equipped to tackle the complexities of this research [21-24].

This review has a few limitations that have been considered when considering the findings. A significant limitation of this review is that the studies identified and included provided very little detail on the health worker’s experiences abroad and the impact of migration on the health sector (if they were mentioned). It will, therefore, be difficult to provide a comprehensive account of the health worker migration situation and its impacts on the health sector in Nigeria. Given the limited information available in the literature, it may be necessary to consider broadening the scope of the review in the future. This could involve incorporating nonacademic literature and reports, a step that could potentially provide a more comprehensive understanding of the situation. Another potential limitation is the exclusion of any language other than English, such as French language studies. This is due to the reviewers’ lack of proficiency in French, making the use of French language studies a time-consuming process. This may have introduced a language bias.

Given the broad scope of the review, it has been a challenge to frame precise research questions and define specific inclusion criteria. This complexity has made it difficult to assess the relevance of the retrieved studies. Moreover, there was significant variation in the concepts and definitions of health professional and “brain drain” among these studies, a factor that will likely influence the findings once the data is extracted and synthesis is carried out.

### Conclusion

This analysis sheds light on the studied topic of health care staff migration and the limited data regarding its extent and effects, despite public worry and ample unofficial publications on the matter. The absence of supporting data has resulted in differing opinions; some term it a “crisis” while others raise concerns. Our scoping review approach has mapped the key concepts and sources, provided an understanding of the existing evidence, and paved the way for a comprehensive follow-up review.

## Abbreviations

JBI: Joanna Briggs Institute
PCC: Population, Concept, Context
PRISMA-ScR: Preferred Reporting Items for Systematic Reviews and Meta-Analyses extension for Scoping Reviews
PROSPERO: International prospective register of systematic reviews

## Data Availability

All supporting data will be reported in the manuscript. Any raw data sets supporting the results will be available and presented in the main manuscript or additional supporting files when submitting the systematic review article.
